# Non-Invasive Strategies for Nose-to-Brain Drug Delivery

**Published:** 2020-12-10

**Authors:** JT Trevino, RC Quispe, F Khan, V Novak

**Affiliations:** Department of Neurology, SAFE Laboratory, Beth Israel Deaconess Medical Center, Harvard Medical School, Boston, MA, USA

**Keywords:** Intranasal administration, Nose-to-brain, Bioavailability, Biodistribution, Devices

## Abstract

Intranasal drug administration is a promising method for delivering drugs directly to the brain. Animal studies have described pathways and potential brain targets, but nose-to-brain delivery and treatment efficacy in humans remains debated. We describe the proposed pathways and barriers for nose-to-brain drug delivery in humans, drug properties that influence central nervous system delivery, clinically tested methods to enhance absorption, and the devices used in clinical trials. This review compiles the available evidence for nose-to-brain drug delivery in humans and summarizes the factors involved in nose-to-brain drug delivery.

## INTRODUCTION

Neurological disorders are the leading cause of disability worldwide, increasing the burden on healthcare [1]. Brain drug delivery is challenging due to the Blood Brain Barrier (BBB), the complexity of the brain, and safety and toxicity concerns [2]. Nose-to-brain drug delivery has emerged as a novel, non-invasive route with advantages over systemic drug administration such as: evasion of systemic toxicity, better side effect profile, non-invasiveness, short latency, and increased Central Nervous System (CNS) bioavailability [3,4]. Nose-to-brain drug delivery bypasses the BBB through neural connections among the olfactory epithelium, olfactory bulb, trigeminal nerve, and the brain [5,6].

This review compiles the available evidence for nose-to-brain drug delivery in humans and provides a framework to determine the feasibility and limitations of this approach. We describe the proposed pathways, potential barriers, optimal drug properties, agents that may promote nose-to-brain delivery, devices targeting this pathway; review the evidence for brain bioavailability and bio distribution, and efficacy evidence from clinical trials.

## METHODOLOGY

A literature search was performed through EMBASE, PubMed-NCBI Database, and Google scholar including published scientific articles in English. EMBASE search criteria: nose: ti, ab, kw and brain: ti, ab, kw and human: ti, ab, kw. Google scholar search criteria: allintitle: nose, brain, delivery. PubMed search criteria: nose-to-brain; intranasal administration; intranasal device; brain targeting; blood-brain barrier; olfactory epithelium; olfactory nerve; trigeminal nerve; physiological barriers; physicochemical properties; absorption enhancers; formulation; pharmacokinetics; bioavailability; bio distribution. Articles that did not measure direct or indirect evidence of nose-to-brain delivery were not considered for this review. 280 articles were screened, 93 excluded because they did not focus on nose-to-brain delivery and 187 papers were included. We included 93 clinical studies, 65 non-clinical studies, 24 reviews, 2 case reports, 1 case series, 1 survey, and 1 patent. Per the Centre for Evidence-Based Medicine (CEBM) Levels of evidence [7], studies were classified as Level 2 evidence if they mentioned random treatment allocation in their study design or if observational with a dramatic effect, Level 3 if they were non-randomized controlled studies, and Level 4 if they were presented as a case report or case series.

## PATHWAYS FOR NOSE-TO-BRAIN DRUG DELIVERY

**Figure 1:**
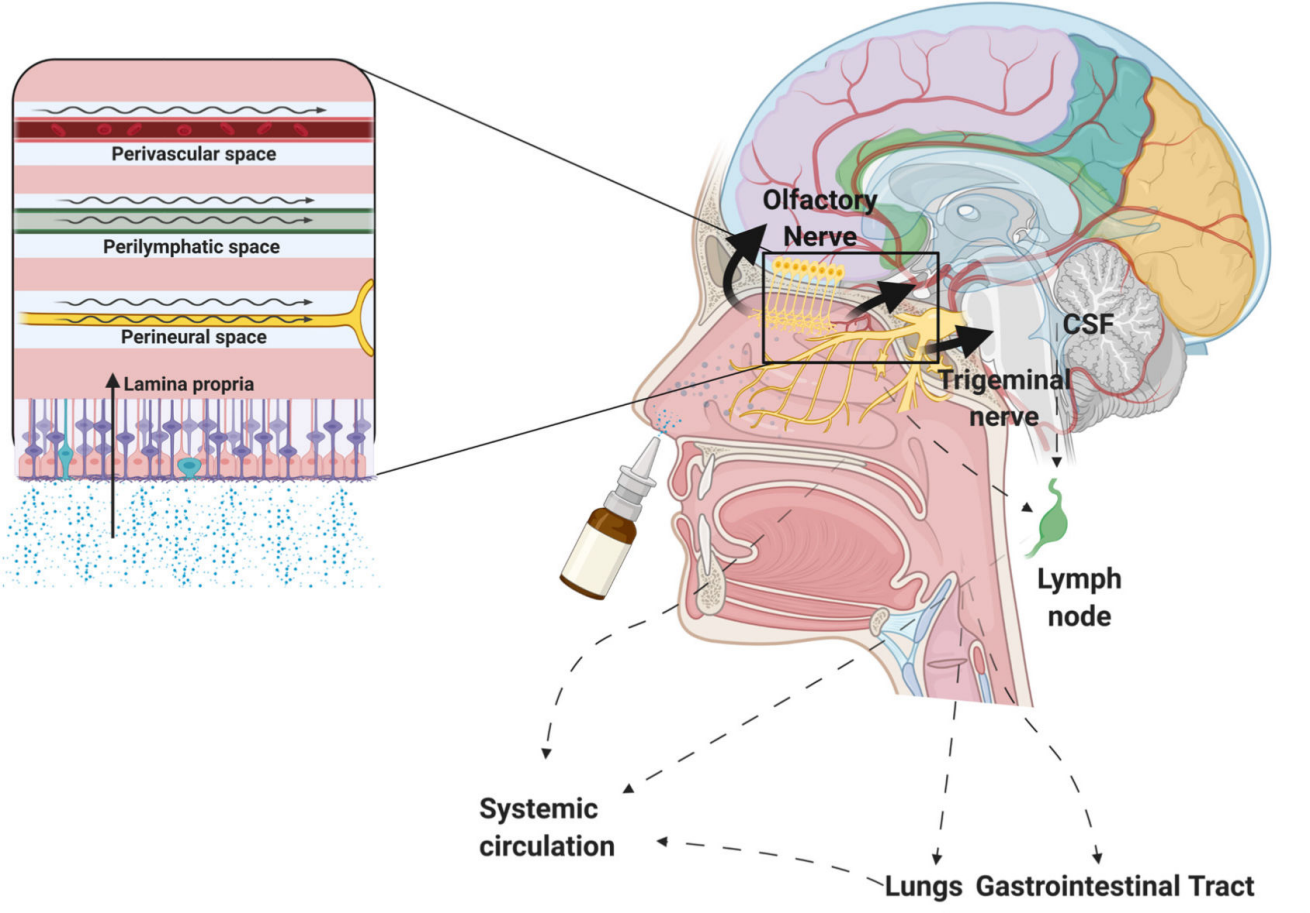
Nose-to-brain delivery pathways. The target region for effective nose-to-brain drug delivery is the olfactory epithelium in the upper nasal cavity. This region contains olfactory nerve cells which bypass the BBB & provide direct access to the brain & CSF. Nose-to-brain transport is depicted by the solid lines; clearance is depicted by the dotted lines. The box shows transport through the following routes: perivascular pump, bulk flow, lymphatic drainage, & endoneural transport through the olfactory & trigeminal nerves. Minimal amounts of intranasally administered drug may enter the CNS via carotid artery branches; the main limiting barrier for this route is vascular endothelium permeability. Systemic absorption through the nasal mucosa is not significant.

The nasal cavity is divided in half by the nasal septum; each half has three regions; the nasal vestibule, the respiratory region, and the olfactory region. The nasal vestibule is the entrance to the nose; it is lined with squamous epithelium and contains hair (vibrissae) and sebaceous glands [8]. The respiratory region constitutes most of the nasal surface area. It is lined with ciliated pseudostratified columnar epithelium (respiratory epithelium) and contains the nasal turbinates. The nasal turbinates are vascular structures containing sinusoids and erectile tissue they humidify and warm incoming air and allow for venous congestion. The olfactory region, is located in the roof of the nasal cavity, approximately 7-cm away from the nostrils. It is lined with pseudostratified columnar epithelium (olfactory epithelium), and contains the olfactory nerve which provides direct CNS access by bypassing the BBB Figure 1.

Several pathways for human nose-to-brain delivery have been proposed based on pre-clinical studies. Evidence from animal studies is not readily transferable to humans due to fundamental anatomical and physiological differences. Nevertheless, clinical trials have demonstrated nose-to- brain delivery in humans; but the pathways have not been confirmed.

Once inhaled, substances enter the nasal vestibule where vibrissae, turbulence, and mucosal contact filter particles larger than 12 μm [8]. Substances pass through the nasal valve, composed of the nasal turbinates and cartilages, and arrive to the respiratory region. The nasal turbinates undergo alternating congestion and decongestion every 3–7 hours due to selective autonomic innervation [8]. Age and increased tissue elasticity can result in temporary nasal valve collapse [8]. The nasal valve has the smallest cross-sectional area of the nose and small changes in this area are likely to affect air flow. This mechanism reduces the amount of substances that reach the olfactory region. However, up to 45% of a drug can be delivered into the olfactory region with special devices [9]. The remaining drug may be absorbed in the respiratory region, which has the largest nasal surface area (around 130 cm^2^), and a rich vascular supply [10]. The maxillary branch of the trigeminal nerve innervates the respiratory region and enters the CNS through the pons; making it a relevant target for CNS drug transport [10,11]. A recent study in rats has shown that intranasal insulin can reach the CNS alongside the extracellular components of the trigeminal nerve [12]. These findings suggest that intranasally administered macromolecules can bypass the BBB and enter the CNS along the trigeminal nerve [13].

After overcoming the nasal valve, drugs enter the olfactory region, the only place where the brain meets the outside world. The olfactory epithelium has been proposed as the predominant site of drug absorption for nose-to-brain delivery [14].

The surface area of the human olfactory region is between 2–10 cm^2^. However, the olfactory nerve can potentially be accessible over a larger area [15]. Once a drug crosses the olfactory epithelium, intracellular and extracellular transport ensues along the olfactory nerve. Transport occurs through paracellular passive diffusion for lipophilic drugs and carrier-mediated transport for hydrophilic drugs; endocytosis and axonal transport play a smaller role [14].

Olfactory nerve cells penetrate the cribriform plate of the ethmoidal bone and project to the olfactory bulb in the CNS. The olfactory bulb relays sensory information to the amygdala, orbitofrontal cortex, and hippocampus. In the olfactory bulb, the drug can enter the brain through axonal transport, passive diffusion, or carrier-mediated transport depending on the drug’s characteristics. The extracellular pathway involves absorption through the paracellular space of the olfactory mucosa, into the lamina propria, and the Cerebrospinal Fluid (CSF) through perivascular and perineural transport [3]. In the lamina propria, the drug undergoes different transport mechanisms along the nerves, vessels, and lymphatics, namely intracellular and extracellular transport, perivascular pumping, and bulk flow.

Bulk flow and perivascular pumping within the lamina propria, a subepithelial layer of loose connective tissue containing nerves, blood vessels, and lymphatics, have also been shown to deliver substances into the brain parenchyma [16]. The perivascular pump mechanism depends on systolic arterial pressure waves travelling across the vessels which compress the perivascular space and help move its contents forward [10]. The nose has a rich vascular supply from ethmoidal arteries branching from the ophthalmic and internal carotid artery [8]. Drugs may travel through the perivascular spaces along these vessels into the CNS [5,11].

Pre-clinical studies comparing intranasal and arterial administration have found that intranasally- administered substances are present in cerebral perivascular spaces within 20 minutes of administration [16], have higher concentrations in the dura mater and circle of Willis [17], and have higher concentrations in deep and superficial cervical nodes of rats; suggesting potential transport through lymphatic drainage from the nasal passages and CSF [17]. Minimal amounts of intranasal drugs enter the CNS via branches of the carotid artery including the maxillary, ophthalmic, and facial arteries. The permeability of the vascular endothelium is the main limiting barrier for this route. Further, the nasal cavity has a rich autonomic innervation; transport along parasympathetic nerves to the sphenopalatine ganglion cannot be excluded.

Drugs unable to reach the olfactory region undergo enzymatic degradation and mucociliary clearance. A small amount of the remaining drug is potentially reabsorbed into the systemic circulation via the respiratory mucosa; although this might not be significant [10].

## OVERCOMING ABSORPTION BARRIERS

Drug formulation is key for safe and effective nose-to-brain delivery, and may determine the absorption pathway it will follow [11,18]. The absorption pathway and molecular weight are related to bioavailability. There is an inverse relationship between molecular weight and percent drug absorption. Nose-to-brain transport depends on the physicochemical characteristics of the drug and the physiology of the human nose. Liquid formulations are well established and have been shown to be more effective for intranasal drug delivery; however they are subject to rapid mucociliary clearance and gravity [9,18].

Physiological barriers for nose-to-brain delivery include the nasal vestibule, nasal valve, epithelial tight junctions, efflux transporters, nasal metabolism, mucociliary clearance, surface area of the olfactory region, presence of drug-specific target receptors/transporters, and the BBB [19–21].

### Permeation enhancers and epithelial tight junctions

The tight junctions of the olfactory and respiratory epithelium and their protective mucus lining act as selective filters that decrease permeability and diffusion [21]. During passive diffusion, drug lipophilicity is paramount; whereas during active transport, a prolonged nasal residence time is crucial [19]. Absorption through the olfactory epithelium is reduced for drugs with molecular weight over 1000 Da due to low permeability and poor absorption through the endothelial basement membrane [9,22]. Permeation enhancers have been tested to improve the absorption of drugs with large molecular weight. Proposed mechanisms include: increased membrane fluidity and tight junction permeability, hydrophilic pore generation, and reduction of viscosity and enzymatic activity [19].

Penetratin, a cell-penetrating peptide, enhanced insulin delivery into the rat brain [23]. Commonly used permeation enhancers include: cyclodextrins, surfactants, saponins, fusidic acids, phospholipids, bile salts, laureth-9-sulfate, and fatty acids [19]. Bioadhesive materials such as carbopol and starch microspheres have also been shown to increase tight junction permeability [24]. Further, mucoadhesive agents such as chitosan have been shown to enhance permeation by opening tight junctions in addition to improving adhesion and prolonging residence time in the nasal mucosa [19].

### Mucoadhesive agents and mucociliary clearance

Mucociliary clearance transports drugs from the respiratory epithelium to the nasopharynx, increasing the risk of entering the gastrointestinal tract. The olfactory cilia are immotile; mucus overproduction results in migration of the mucus layer towards the respiratory region and clearance by respiratory cilia. This mechanism protects against drug inhalation, reduces nasal residence time, and decreases absorption in the respiratory region [18]. Mucociliary transit time in healthy subjects ranges from 2.5 to 25 minutes [19]. Administering compounds with semisolid formulations and mucoadhesive agents may decrease the mucociliary clearance rate and potentially overcome this barrier. Semisolid gels with increased viscosity enhance nasal residence time and brain uptake by up to two-fold [18,25].

Mucoadhesives such as carbopol and starch microspheres enhance absorption by opening intercellular tight junctions and increasing the nasal residence time [3]. Trymethyl chitosan complexes successfully enhanced the nose-to-brain delivery of insulin [26] and buspirone [27] in rats. Tamarind seed polysaccharide has also been shown to enhance selective particle deposition and retention in the olfactory mucosa under simulated conditions using a nasal cast model [28]. Mucoadhesive agents also increase bioavailability for nose-to-systemic drug delivery [29].

### P-glycoprotein efflux transport and nano carriers

P-glycoproteins are glycosylated membrane proteins that act as multidrug resistance pumps across the nasal mucosa and BBB. Intranasally administered drugs are subject to active P-glycoprotein efflux transport [19,30]. Nano carriers are a promising strategy to bypass this barrier [31]. They achieve high efficacy and increased absorption rates by encapsulating and protecting the drug from biological and chemical breakdown [31,32]. Nanostructured lipid carriers have a wide range of uses, have less toxicity, and allow for controlled or sustained release of the drug [32]. The advantages of nanocarriers include: minimum toxicity, biodegradability, physical stability, and compatibility with small molecules, peptides, and nucleic acids [33].

### Nasal metabolism and enzyme inhibitors

Although the nose provides a low metabolic environment, drug metabolism in the nasal cavity is considered a major barrier for nasally-delivered proteins and peptides. Cytochrome-P450 enzymes, exopeptidases, and endopeptidases in the respiratory and olfactory mucosa lead to local enzymatic degradation and potentially limit drug absorption [19,21,34]. Peptidase inhibitors reduce nasal metabolism and prolong residence time, aiding absorption and improving bioavailability [24]. Commonly used enzyme inhibitors includes: bestatin, amastatin, boroleucine, fusidic acids, and phospholipids [19].

## DEVICES FOR NOSE-TO-BRAIN DELIVERY

The nasal vestibule and nasal valve are the first barriers to reach the olfactory region. Drugs delivered with conventional nasal delivery systems deposit here and do not reach the olfactory epithelium [9,35]. Once deposited in the nasal vestibule and turbinates, drugs may be absorbed into the systemic circulation, swallowed, or inhaled. This is undesirable as drug inhalation does not come without risk. The Exubera® trial highlighted the risks of inhaling insulin; the trial was stopped due to hypoglycemia and respiratory adverse events [36].

Studies using human nasal cast models and mathematical algorithms have tried to determine the ideal conditions for olfactory region deposition and nose-to-brain absorption in humans. Results have shown that ideal particle size for olfactory deposition is between 1 nm and 10 μm [37] with a flow rate between 5–20 L/min [38]. Based on these results, new devices have been designed to improve drug deposition into the olfactory epithelium; some of which have been tested in clinical trials. The decisive role that specialized delivery devices play on nose-to-brain delivery was recently highlighted by a recent study in which unreliable device performance required investigators to switch devices mid-study. (Table 1) describes intranasal delivery devices used in clinical trials that have shown promising results in terms of safety and efficacy across different outcome measures. Technical specifications of these devices and findings of included clinical trials are described below.

### ViaNase™

ViaNase™ (Kurve Technology, Inc. Lynnwood, WA, and USA) electronic atomizers create a vortex of nebulized particles to maximize distribution to the upper nasal cavity and minimize pharyngeal deposition. The device allows for precise electronic dosing, targeted delivery into the olfactory epithelium, and maximizes nose-to-brain transport [39,40]. Intranasal Insulin (INI) delivered using ViaNase™ devices has been shown to modify functional connectivity within memory networks [41], improve cortical blood flow [42], enhance vasoreactivity, cognition [43], and improve functionality [4,44], without altering fasting plasma glucose and insulin [45]. This device was used in a subset of 49 participants in the Study of Nasal Insulin to Fight Forgetfulness (SNIFF) trial (NCT01767909), who experienced a modest improvement of verbal recall [46].

However, the investigators switched devices mid-trial, due to frequent malfunction of the trial-specific design modifications. The ViaNase™ device was also used in the Memory Advancement ™ by Intranasal Insulin in Type 2 Diabetes (MemAID) trial (NCT02415556), which evaluated the long term effects of INI on cognition, memory, and gait in older people with type 2 diabetes (results not available) [47].

The ViaNase™ is currently being tested in clinical trials with patients with psychiatric disorders (NCT04071600, not yet recruiting; NCT03943537, ongoing), post-stroke (NCT02810392, completed), and cognitive impairment related to multiple sclerosis (NCT02988401, ongoing).

### Precision olfactory delivery®

The Precision Olfactory Delivery® (Impel Neuropharma, Seattle, WA, USA) device features a semi-disposable unit-dose format, vowing consistent dose delivery, and higher CNS bioavailability when compared to systemic administration. This device uses an inert liquid (hydrofluoroalkane) that forms a gas propellant to deliver liquids and powders to the olfactory epithelium [48]. This device has been shown to deliver up to 45% of the administered dose to the upper nasal cavity [49]. Studies using this device have demonstrated a higher deposition of radiolabeled drug compounds in the olfactory region of rats and a higher drug concentration visible in human brain regions [50,51]. The device was used in 240 participants of the SNIFF trial [46], but did not show improvement of memory in patients with mild Alzheimer’s disease. The Precision Olfactory Delivery® device was used in recently completed clinical trials (results not available) in patients with migraines (NCT03557333), Parkinsons Disease (PD) (NCT03541356), investigating memory in healthy participants (NCT02758691), and safety of intranasal olanzapine (NCT03624322).

### Aero Pump system

The Aero Pump system for nasal application (Aero Pump, Hochheim, Germany) has been used for INI administration. This device uses a mechanical spring with an integrated backflow block to deliver drugs and prevent contamination. Systematic reviews [52,53] have assessed the effects of INI and melanocyte-stimulating hormone/adrenocorticotropin4–10 (MSH/ACTH4–10), a melanocortin receptor agonist, on memory, cognition, and weight regulation using this device. INI has shown promising effects on memory and MSH/ACTH4–10 on weight loss in non-overweight subjects. Several double-blind Randomized Controlled Trials (RCT) have administered INI using this device to assess its effect on weight by modifying cerebral energy metabolism [54], branched-chain amino acid levels [55], and regional blood flow to the insular cortex [56]. These studies support the hypothesis that brain insulin has a role in coordinating energy intake, metabolism, and cerebral blood flow in regions that control eating behavior. However, INI did not show improvement of memory performance in one trial [57]. Another trial investigated the effect of INI on tissue-specific insulin sensitivity (NCT02933645) (results not available).

### Metered nasal dispenser

The metered nasal dispenser (Pharmasystem, Markham ON, Canada) is a finger actuated device that can deliver 25–200 μl (median: 100 μl) per spray. It can be used in any position and is suitable for daily drug administration over an extended period. When delivered with this device, drugs with a narrow therapeutic window demonstrate lower efficacy [39]. Recent studies using this dispenser found that INI reduced endogenous hepatic glucose production [58,59], suggesting peripheral effects rather than central effects. Ongoing clinical trials are investigating the effect of INI on blood glucose, plasma and CSF insulin concentrations (NCT02729064), post-operative delirium (NCT03415061), and post-operative cognitive function (NCT03324867).

### Mistette MK Pump II, GL18

The Mistette MK Pump II, GL18 (MeadWestvaco Calmar, Hemer, Germany) uses a mechanical spring to produce a fine mist. One RCT used this device to administer INI to the brain and assessed the effect on pancreatic glucose and the results suggested brain-pancreas crosstalk [60].

### SP270+

The SP270+ (Nemera, la Verpilliére, France) has an actuator that produces droplets with a median size of 40 μm and an elliptical plume. The SP270+ was recently used in a double-blind, randomized, crossover, fMRI study investigating the effect of INI on cerebral blood flow; which demonstrated changes in blood flow after INI delivery against placebo [61]. A pre-clinical study compared this device and the VP3 device and concluded that both produced similar sized droplets (mean volume diameter 40.8 ± 8.9 μm and 42.4 ± 2.8 μm, respectively). However, the SP270+ was negatively affected by viscosity variations.

### OptiMist™

The Optimist™ (OptiNose AS, Oslo, Norway) device is activated by blowing into a mouthpiece to close the soft palate and isolate the nasal cavity while providing positive pressure. This mechanism minimizes the risk of lung deposition during nasal administration [62] and optimizes delivery into the olfactory epithelium [63]. This device has been primarily tested for local nasal drug delivery (nasal polyposis, sinusitis) and to a lesser extent migraine (NCT01507610), headache (NCT01667679), and autism spectrum disorder treatments (NCT02414503).

Optimist™ has been reported to deliver up to 18% of the dosage to the upper nasal cavity [49]. A comparative study using a human nasal cavity replica found this device performed significantly better than a regular aerosol mask in delivering particles to the olfactory region [37]. A double-blind RCT using Optimist™ to deliver midazolam and sumatriptan nasal formulations in adults showed no serious adverse events and suggested drugs could be delivered directly into the brain through routes that bypass the BBB [64,65].

### Unit Dose system

Unit Dose system (Aptar Pharma, Crystal Lake, IL, USA) was designed to address the nose-to-brain pathway. This device uses a piston with a ball-valve at the tip to deliver drugs. It features one-handed actuation and is suitable for liquid and powdered drug delivery [39]. Merkus et al. used this device to administer peptide drugs to neurosurgical patients with a CSF drain and failed to demonstrate nose-to-brain drug delivery [66]. Unit Dose System was used in a RCT, which evaluated the safety and efficacy of three doses of a third-generation calcitonin gene-related peptide receptor antagonist known as BHV-3500 (vazegepant) for acute treatmentof moderate to severe migraine (NCT03872453) [67]. Preliminary results showed a reduction of migraine symptoms when compared to placebo.

### Sipnose

The Sipnose device (SipNose LTD, Yokneam, Israel) uses a pressurized delivery system with compressed air, resulting in an aerosol with a narrow plume geometry which targets the olfactory epithelium. Its mechanism allows better localization of aerosolized drug in the olfactory epithelium and the trigeminal nerve. This device can be used with liquids, dry powders, and molecules of small and large sizes [68]. The Sipnose device is currently being used in clinical trials looking at preoperative anxiety and sedation in infants (NCT03635398, not yet recruiting) and safety of INI in type 1 diabetes patients (NCT04028960).

## NOVEL DEVICES NOT YET USED IN CLINICAL TRIALS

### Naltos™

The Naltos™ (Nanomerics, London, UK) is a single-use, disposable device that uses an inert gas to propel powder through the nares [69]. Developers intend to use this device for delivering medications for postoperative and neuropathic pain, among others [70]. Testing is still at the pre-clinical stage.

### VP3

The VP3 device (Aptar Pharma, Le Vaudreuil, France) has high dose accuracy and is suitable for administering suspensions and viscous formulations. This device coupled with the Aptar 144GI actuator generated a minimal amount of droplets that could be potentially deposited in the lower airways (3% of droplets <10 μm) [71]. It was compared to the SP270+device and results showed they produced similar-sized droplets and the VP3 was better at handling viscous solutions than the SP270+; Results warranted testing at the pre-clinical level.

### Aeroneb^®^ Pro

The Aeroneb^®^ Pro (Aerogen 112 Inc. Galway, Ireland) is a reusable nebulizer that produces a fine particle, low-velocity aerosol used to deliver drugs systemically. This device has been tested in human nose models, which showed it has the technical capabilities to be used as a nose-to-brain delivery platform [38].

### Versidoser^®^ and VRX2™

The Versidoser^®^ (Mystic Pharmaceuticals, Austin, TX, USA) is designed to deliver liquids using nozzle dispensing technology. According to its manufacturer, it allows for precise, efficient, and safe dosing of liquids across a wide range of volumes and fluid properties [72]. The VRX2™ uses the same technology and mechanism to dispense powders and reconstituted combination liquids. Developers recently obtained a patent for dose dispensing containers which have been specifically designed to target nose-to-brain delivery and will be incorporated into the Versidoser® and VRX2™ to extend their capabilities [73].

## BRAIN BIOAVAILABILITY AND BIODISTRIBUTION AFTER NOSE-TO BRAIN DELIVERY

Research concerning bioavailability and biodistribution following intranasal drug delivery has relied on preclinical animal studies [74–76], use of human nasal replica casts, mathematical modeling, imaging, and in a much smaller scale, human CNS/CSF sampling.

Brain bioavailability, biodistribution, and the efficacy of nose-to-brain delivery are determined by dynamic and concurrent biological factors and processes. Pre-clinical studies have provided evidence of drug activity in the brain following intranasal administration [12,16,17,76–78]. To date, the most extensive, descriptive, and quantitative pre-clinical study of in vivo brain targeting efficiency via the nasal route analyzed 73 publications and 82 compounds. This study showed intranasal administration is more efficient than systemic administration [75], confirming the feasibility of in vivo nose-to-brain drug delivery in animals. An extensive review of pharmacokinetic preclinical studies comparing the Area Under the Curve (AUC) of brain tissue and CSF showed higher brain bioavailability for a broad range of drugs [75]. One study measuring concentrations in rat CSF after intranasal administration resulted in a relative bioavailability (AUC intranasal/AUC intra-arterial) of 43% for procaine and 100% for tetracaine, bupivacaine, and lidocaine [74]. Intranasal administration of remoxipride in rats showed a total bioavailability of 89%, out of which 75% was attributed to nose-to-brain transport [76]. Nevertheless, qualitative and quantitative differences between animal and human nasal surface area, olfactory region, capillaries, airflow rate, cerebral blood flow, CSF turnover, brain tissue binding, and intracerebral distribution, may be a limitation for successful translation of preclinical evidence [15,79,80].

Brain imaging can be used as an alternative to brain sampling to determine nose-to-brain delivery and its effectiveness in clinical and preclinical trials [81]. MRI, Positron Emission Tomography (PET), Single-Photon Emission Computed Tomography (SPECT), and gamma scintigraphy have been used in clinical studies. Several trials using different drugs and devices have demonstrated human nose-to-brain delivery through changes in brain metabolism [44,82], selective insulin impairment [83], changes in brain blood flow [61], antineoplastic effects [84], neuromodulation [85], and brain bioavailability and biodistribution [86,87]. The use of in vivo imaging techniques can ease the translation of intranasal drug delivery from animals to humans [81]. PET/MRI is hypothesized to be the most sensitive method to quantify in vivo nose-to-brain delivery, as it provides high tissue contrast and good spatial resolution [81].

Evidence of nose-to-brain delivery in humans has also been obtained from comparing concentrations of melanocortin, vasopressin, and insulin in CSF and systemic circulation after intranasal administration in healthy volunteers [88]. Post INI administration, CSF insulin levels increased within 10 minutes, peaked between 30 and 45 minutes, and remained elevated at 80 minutes [88]. This timeline was later replicated by other clinical and animal studies [16,17,45]. Human nose-to-brain transport has been questioned by some studies [89]. A cohort (n=8) of neurosurgical patients with CSF drains received intranasal and intravenous melatonin and hydroxycobalamin; CSF and plasma comparisons failed to demonstrate nose-to-brain drug transport. These findings were attributed to the use of non-peptide drugs (which are better absorbed by the systemic circulation), and low doses of the administered intranasal drug (100 μL per nostril) [66]. Further, studies have shown that nose-to-brain delivery is particularly sensitive to methodological variation, which could also explain these findings [57].

## CURRENT CLINICAL EVIDENCE

Tables 2 and 3 summarize clinical trials that looked at direct and indirect evidence of nose-to-brain delivery.

Tables 2 and 3 summarize clinical trials that looked at direct and indirect evidence of nose-to-brain delivery.

### Insulin

INI is the most widely tested drug in RCTs for nose-to-brain delivery due to its potential for improving memory, cognition, and appetite control (Table 2). Even though insulin has a high molecular weight (5808 Da), studies have shown peptide molecules can be absorbed through specialized pathways involving receptor-mediated transcytosis and passive diffusion [88,90–92]. Moreover, the presence of insulin receptors in the olfactory bulb, hippocampus, hypothalamus, and lower brainstem, makes it an ideal candidate for nose-to-brain delivery [93].

RCTs have demonstrated successful nose-to-brain insulin delivery through the use of fMRI [42,57,94,95], cerebral blood flow measurements [42,56,83], CSF measurements [88], functional disability scales [64,65], and cognitive tests in healthy, diabetic, and Alzheimer’s disease populations [43,44,52,96]. Studies have also demonstrated INI increases cerebral metabolism [54], affects brain-pancreas crosstalk [60], lowers endogenous glucose production [59], and has no effects on triglyceride secretion and lipid content [55,58].

Most RCTs using INI have administered doses of 40 and 160 International Units (IU) and have achieved short term efficacy without any major adverse events [4,21,41–43,54,56,58,97]. One study comparing intranasal administration of 10 IU, 20 IU, 40 IU, and 60 IU of insulin, demonstrated improved verbal memory in their study population with a performance peak at 20 IU [45].

Administering 20 IU twice daily may affect efficiency by increasing exposure duration (as opposed to one 40 IU dose) while maintaining the same dose [45].

### Cholecystokinin (CCK)

CCK has been administered intranasally to test cognitive, behavioral, motor, and physiological outcomes in healthy, young adults [98–100]. Pre-clinical studies have shown varied results regarding successful nose-to brain delivery of CCK.

Nose-to-brain CCK delivery in humans has been demonstrated by studies observing increases in event-related potentials in the brain following intranasal CCK [101–103]. One study described a maximum recording 120 minutes following administration and another noted no dose-response relationship of CCK after administering 10 and 20 micrograms [102,104]. Repetitive intranasal administration favors bypassing a saturable dose-response curve and enhances effectivity [104]. A study involving PD patients observed delayed brain potential signals following intranasal CCK, possibly explained by the effect of the neuropeptide on transmitter systems (e.g. GABAergic) rather than the dopamine system [100].

### Erythropoietin (EPO)

EPO has been tested in the setting of preventing amyloid toxicity in Alzheimer ‘s disease and as a neuroprotective factor in stroke [105–109]. A phase I human study showed EPO to be safe, well tolerated, and did not stimulate erythropoiesis in healthy volunteers [110]. Further clinical studies in humans are required to establish efficacy in treating CNS diseases.

### Melanocortin

Melanocortin has been used to promote lipid metabolism and decrease body fat in animals and humans [111–113]. A direct effect of melanocortin in human CNS has been suggested. An experiment conducted observing changes in melanocortin CSF levels following intranasal administration found higher levels 80 minutes after administration compared to placebo [88]. An increase in CSF concentration with higher doses of intranasal melanocortin [88] was also observed. A clinical trial saw increased abdominal lipolysis in adipose tissue 45 minutes after melanocortin receptor agonist administration against placebo [114]. Reductions in body fat, weight, plasma leptin, and insulin levels were demonstrated following intranasal melanocortin administration in humans [111].

### Glutathione

Glutathione deficiency in the brain has been reported in several disease states including Parkinson Disease (PD) [115]. One study administered intranasal glutathione in patients with PD and followed levels in the CSF using Magnetic Resonance Spectroscopy (MRS) and found significantly higher levels compared to baseline for most time points [116]. A phase I study did not find differences among safety measures comparing intranasal glutathione to placebo in PD patients [117] (NCT01398748). Further, a survey of intranasal glutathione administration in PD patients showed most respondents found the therapy effective and without significant adverse events [118]. A Phase IIb study in PD patients showed improvement in Unified PD Rating Scale and motor subscore over three months of medium-dose intranasal glutathione treatment compared to baseline [119]. However, they found neither the low or medium-dose treatment group to be superior to placebo [119]. Further studies are warranted to understand the role of intranasal glutathione therapy in patients in a deficient state.

### Perillyl alcohol

Perillyl alcohol is a potent antitumor agent used for the treatment of recurrent gliomas [120]. Phase I studies have administered the medication orally have not shown promising results [121,122]. Intranasal administration of perillyl alcohol in humans was first described in a case report of a patient with anaplastic oligodendroglioma intranasal treatment resulted in tumor shrinkage [123]. Multiple trials have been successful at treating multiple gliomas, anaplastic oligodendrogliomas, astrocytomas, and recurrent glioblastomas with intranasal perillyl alcohol [124–126]. The ineffectiveness of perillyl alcohol as an oral agent and its subsequent effectiveness when administered intranasally suggests the drug can enter the BBB via previously mentioned pathways including the olfactory and trigeminal nerve.

### Angiotensin II

Angiotensin II has been administered intranasally to test cardiovascular control [127]. Pre- clinical studies showed similar changes in blood pressure and norepinephrine levels after comparing intranasal and intra-cerebroventricular administration of angiotensin II, suggesting successful nose-to-brain delivery [128,129]. Nose-to-brain delivery of angiotensin II was clinically tested by administration following blockade of peripheral receptors [130]. Interestingly, results showed increased levels of plasma angiotensin II, unaffected plasma levels of vasopressin and norepinephrine, and an acute reduction in blood pressure [130]. These outcomes demonstrate opposite findings when compared to no peripheral blockade of receptors, indicating a need for further research to understand the role central angiotensin II plays in blood pressure regulation.

### Neurotrophic factors

Successful nose-to-brain delivery of neurotrophic factors has been demonstrated in animal models [17,131–138]. Human trials with neurotrophic factors are lacking and evidence is limited to case studies. One pilot study administered intranasal nerve growth factor over 12–18 months to two females with frontotemporal dementia and showed a slower decline measured by clinical and neurological outcomes [139]. Another case study administered intranasal nerve growth factor for 10 days in a four-year-old boy in a persistent unresponsive wakefulness syndrome following a traumatic brain injury [140]. Following administration, CSF nerve growth factor levels were increased [140]. Clinically, there were improvements in voluntary movements, facial mimicry, phonation, attention, verbal comprehension, ability to cry, cough reflex, oral motility, feeding capacity, bowel and urinary function [140]. More clinical studies are warranted to investigate the feasibility of intranasal delivery of neurotrophic factors.

## CONCLUSION

Safe and effective nose-to-brain delivery has been shown by direct and indirect measurements in pre-clinical and clinical studies. Three main pathways for nose-to-brain delivery have been proposed and supported by variable evidence: olfactory nerve, trigeminal nerve, and perivascular transport. Physicochemical drug properties, physiological barriers, delivery devices, and even head positioning may influence the efficacy of drug delivery into the brain. The advent of new nose-to- brain delivery technologies, including devices and drug formulations, and the improvement of the currently available ones may improve overall nose-to-brain delivery. These technologies will help broaden and exploit the therapeutic potential of this pathway and may shift the current paradigm of neurodegenerative diseases. Insulin is the most widely studied drug for nose-to-brain delivery and, there is significant level 2 and level 3 evidence suggesting insulin and other substances can be delivered directly into the brain through the aforementioned pathways. Limitations of studies evaluating other substances are mainly due to lack of randomization, blinding, or case studies. Future clinical studies are needed to determine optimal strategies based on drug dose, formulation, devices, and timing for nose-to-brain delivery. Additionally, clinical investigators should continue to rely on pre-clinical translational pharmacokinetics-pharmacodynamics modeling to improve the safety and effectiveness of the clinical studies they design.

## Figures and Tables

**Table 1:** Specialized delivery devices used in randomized clinical trials Table 1 describes clinical trials that used specialized intranasal delivery devices for nose-to- brain transport. Clinical evidence is further described in Tables 2 & 3.

Author (Year)	Drug (dose)		N participants
**ViaNase**™ (Kurve Technology, Inc. Lynwood, WA, USA) creates a vortex of nebulized particles targeting the olfactory region, maximizes IN distribution, & minimizes pharyngeal deposition.			
Craft et al. (2012) [82]	INI (20 IU & 40 IU)		104
Novak et al. (2014) [43]	INI (40 IU)		29
Zhang et al. (2015) [41]	INI (40 IU)		28
Akintola et al. (2017) [42]	INI (40 IU)		19
Craft et.al. (2017) [44]	INI (40 IU)		36
Craft et al. (2020) [46]	INI (40 IU)		49
		**Total**	**265**
**Precision olfactory delivery**® (Impel Neuropharma, Seattle, WA, USA) uses a gas propellant to deliver liquids & powders to the olfactory epithelium.			
Craft et al. (2020) [46]	INI (40 IU)		240
		**Total**	**240**
**AeroPump** (Aero Pump, Hochheim, Germany) spring mechanism with integrated backflow block to deliver drugs & prevent contamination.			
Schmidt et al. (2009) [141]	INI (0.5–1.5 IU/kg/day)		6
Jauch-Chara et al. (2012) [54]	INI (40 IU)		15
Schilling et al. (2014) [56]	INI (40 IU)		48
Brunner et al. (2016) [57]	INI (40 IU)		11
Scherer et al. (2017) [55]	INI (160 IU)		20
Rodriguez-Raecke (2018) [142]	INI (40 IU)		30
		**Total**	**130**
**Metered Nasal Dispenser** (Pharmasystem, Markham ON, Canada) delivers 25–200 μl (median: 100 μl)/spray; well-suited for daily administration over extended durations.			
Dash et.al.(2015)[59]	INI (40 IU)		8
Xiao et.al.(2017)[58]	INI (40 IU)		9
		**Total**	**17**
**Mistette MK Pump II, GL18** (MeadWestvaco Calmar, Hemer, Germany) spring mechanism produces a fine mist to deliver drugs into the olfactory region.			
Stockhorst et.al.(2011)[143]	INI (120 IU)		32
		**Total**	**32**
**SP270+** (Nemera, La Verpillière, France) an actuator produces droplets (median size: 40 μm) & an elliptical plume to deliver compounds to the olfactory region.			
Wingrove et.al.(2019)[61]	INI (160 IU)		16
		**Total**	**16**
**OptiMist**™ activated by blowing into a mouthpiece to close the soft palate & isolate the nasal cavity while providing positive pressure; minimizing the risk of lung deposition & optimizing delivery into the olfactory epithelium.			
Luthringer et.al.(2009)[144]	Sumatriptan (10, 20 mg)		12
Djupesland et.al.(2010)[65]	Sumatriptan (10, 20 mg)		117
		**Total**	**129**

**ABBREVIATIONS:** IN: Intranasal; INI: Intransal Insulin; IU: International Units

**Table 2:** Clinical trials evaluating nose-to-brain delivery of intranasal insulin.

Author (Year)	Design	N (Male)	Characteristics	Dose	Outcome measures	Conclusion	Evidence level*
Kern et al. (1999) [145]	Double-blind, placebo-controlled, crossover	18 (M)	Male, healthy, 18–34 yo, BMI 23.8 ± 1.2, non-smoking, no history of DM	20 IU	AERP, BP, serum insulin, blood glucose	INI reduced amplitudes of N1 & P3 components of AERP & increased P3 latency. No changes in serum insulin or glucose.	2
Born et al. (2002) [88]	Open label	36 (27 M)	Healthy, 25–41 yo	40 IU	CSF, blood glucose	Increased CSF concentration within 10 minutes of INI administration, peaked at 30 minutes, remained elevated at 80 minutes. No change in plasma glucose.	2
Benedict et al. (2004) [146]	Double-blind, placebo-controlled, parallel	38 (24 M)	Healthy, 18–34 yo, normal weight	160 IU	Blood glucose, insulin, declarative memory, attention, mood	Blood glucose & plasma insulin did not differ compared to placebo. Delayed word-recall improved after 8 weeks. Subjects on INI reported enhanced mood & self-confidence. No systemic side effects.	2
Hallschmid et al. (2004) [147]	Double-blind, placebo-controlled, crossover	40 (24 M)	Healthy, 23–27 yo, normal weight, non-smoking	160 IU	Anthropometry, BIA, WC, eating behavior, hunger, HR variability, epinephrine & norepinephrine levels, plasma ACTH, BP, serum electrolytes, total cholesterol, HDL, LDL, TG	Men on INI lost 1.28 kg body weight, 1.38 kg body fat, WC decreased by 27%. Women on INI did not lose body fat, gained 1.04 kg extracellular water.	2
Benedict et al. (2005) [148]	Double-blind, placebo-controlled, crossover	32 (16 M)	Healthy, 24–25 yo, BMI<25, non-smoking	160 IU	BP, HR, muscular sympathetic nervous activity	After immediate INI, systolic, diastolic, & mean BP increased; muscular sympathetic nervous system activation & HR unaffected.	2
Reger et al. (2006) [149]	Randomized, placebo-controlled, counter-balanced	61 (28 M)	35 healthy; 25 probable AD/aMCI; 68–83 yo	20 IU 40 IU	Blood glucose, insulin, verbal declarative & visual working memory, selective attention, ApoE genotype	No INI effect on plasma insulin or glucose. INI improved story recall, had no effects on attention or working memory. Cognitive responses to acute INI may vary according to APOE genotype.	2
Benedict et al. (2007) [53]	Double-blind, placebo-controlled, crossover	36 (M)	Male, 18–35 yo, BMI<25	160 IU	Plasma glucose, serum insulin, immediate & delayed word recall	Plasma glucose & serum insulin were not affected by acute or sub-chronic INI. Memory performance improved significantly after 8 weeks. Healthy controls did not benefit from acute INI. Insulin aspart has greater potential to improve memory in humans.	2
Benedict et al. (2008) [150]	Placebo-controlled, crossover	32 (14 M)	Healthy, 21–23 yo, normal weight	160 IU	Hippocampus-dependent object location, mirror-tracing, & working memory task, food intake, serum insulin, C-peptide, cortisol, adiponectin, leptin	Hippocampus-dependent memory & working memory improved in women; independent mirror-tracing task was not affected. INI decreased food intake in men, not women. Men did not benefit from INI. Plasma glucose & C-peptide decreased after INI; circulating insulin, cortisol, leptin, & adiponectin not affected.	117
Böhringer et al. (2008) [151]	Double-blind, placebo-controlled, parallel	26 (M)	Male, 20–31 yo, BMI 21–23	40 IU	Baseline plasma & salivary cortisol, HR, BP, & post social stress test	INI effectively lowers stress-induced HPA axis responsiveness.	2C
Hallschmid et al. (2008) [52]	Randomized, placebo-controlled	30 (M)	Male, 31–35 yo, obesity, non-smoking	160 IU	Anthropometry, BIA, WC, HR variability, leptin, insulin, ACTH, cortisol, epinephrine, norepinephrine, declarative & non-declarative memory, mood, selective attention, hunger, thirst, tiredness	INI did not induce body composition or body weight changes. SBP elevated only after initial 40 IU dose, DBP & all other parameters unchanged. Plasma ACTH decreased during INI treatment, INI decreased serum cortisol. Leptin, blood glucose, plasma insulin, epinephrine, & norepinephrine unaffected. Acute INI decreased introversion & anxiousness. Word delayed-recall enhanced after 8 weeks INI.	2
Reger et al. (2008) [45]	Randomized, counter-balanced	92 (NA)	59 cognitively normal, 33 probable AD/aMCI, 70–78 yo	10 IU 20 IU 40 IU 60 IU	Declarative memory, selective attention, visual working memory, psychomotor processing speed ApoE4, blood glucose, insulin, amyloid-β	INI did not affect peripheral glucose or insulin, facilitated verbal memory in memory-impaired adults who were not ApoE4 carriers. 10, 20, & 40 IU improved declarative memory in memory-impaired ApoE4 carriers. INI modulated plasma amyloid-β, acute clinical benefits of treatment greatest with 20 IU.	2
Reger et al. (2008) [96]	Randomized, double-blind, placebo-controlled	25 (NA)	Adults, AD/aMCI, 77–80 yo	40 IU	Story recall, selective attention, response inhibition, fasting glucose, insulin, β-amyloid, cortisol-binding globulin	INI was well tolerated, reduced postprandial insulin levels, improved cognition, & modulated plasma β-amyloid levels.	2
Schmidt et al. (2009) [141]	Randomized, double-blind, placebo-controlled	6 (2 M)	Children, 22q13 deletion syndrome, 9mo-6 yo	0.5–1.5 IU/kg/day	Anthropometry, blood glucose, cortisol, insulin antibodies, neurodevelopmental exam, EEG	1 week: decreased restlessness, improved attention span. 6 months: improved control & coordination of fine & gross motor function, improved everyday life behavior control. 12 months: Improved strength, motor function, speech understanding, use of communication devices, hand function, autonomy, & prolonged attention span.	2
Guthoff et al. (2010) [95]	Randomized, blinded, placebo-controlled, crossover	9 (5 M)	24.6 ± 1.3 yo, BMI 21.4 ± 0.7, HbAlc 5.2 ± 0.1%	160 IU	3T-fMRI during visual recognition task, plasma glucose, insulin, C-peptide, cortisol	Fasting plasma insulin & glucose did not differ between INI-placebo. Reduced cortical activity during food picture categorization after INI; effect restricted to food pictures; placebo had no effect. INI downregulates brain activation by food pictures.	2
Krug et al. (2010) [152]	Double-blind, balanced, crossover	14 (0 M)	Female, healthy, 51–62 yo, BMI 23.7±0.6, postmenopausal	160 IU	Working memory, visuospatial memory, food intake	INI did not affect food intake; enhanced performance in prefrontal cortex-dependent working memory.	3
Stingl et al. (2010) [153]	Randomized, single-blind, placebo-controlled, crossover	20 (6 M)	24–28 yo, 10 BMI 21 ± 0.4 10 overweight/obesity BMI 29 ± 3	160 IU	MEG recordings, functional connectivity analysis, plasma glucose, insulin, & C-peptide	Systemic metabolic parameters did not show significant change after INI. INI modifies global brain network during resting state.	2
Benedict et al. (2011) [154]	Double-blind, placebo-controlled, balanced, crossover	19 (M)	Male, healthy, 18–26 yo, normal weight	160 IU	Energy expenditure, blood glucose, insulin, C-peptide, FFA	INI increased postprandial energy expenditure & decreased postprandial circulating insulin & C-peptide; postprandial plasma glucose did not differ from placebo. INI induced a transient decrease in prandial serum FFA.	3
Fan et al. (2011) [155]	Double-blind, placebo-controlled	30 (10 M)	18–65 yo, schizophrenia, stable antipsychotic dose	40 IU	Serum insulin, plasma glucose, immediate & delayed recall, sustained attention	Decrease in serum insulin after INI, no plasma glucose changes; single dose INI had no significant effect on cognition & is safe in this population.	2
Guthoff et al. (2011) [156]	Randomized, single blind, placebo-controlled, crossover	20 (6 M)	24–28 yo, 10 BMI 20.9±0.4 10 BMI 28.8±0.6	160 IU	HbA1c, fasting plasma glucose, insulin, C-peptide, one-back visual memory task	INI had no effects on blood glucose, insulin, or C- peptide. INI increased components of evoked fields related to identification & categorization of pictures in lean subjects. INI did not modulate food-related brain activity in obese subjects.	2
Stein et al. (2011) [157]	Randomized, open-label, placebo-controlled	32 (15 M)	Community dwelling, ≥60 yo, MMSE 12–24	240 IU	ADAS-Cog, WMS-RLM, MMSE, DAD, GDS, plasma calcium, albumin, uric acid, creatinine	No significant difference between INI & placebo for any endpoint.	2
Stockhorst et al. (2011) [60]	Randomized, double-blind, placebo-controlled	32 (M)	Male, 24.2±0.5 yo, BMI 22.4±0.3	20 IU	Blood glucose, insulin, epinephrine	Blood glucose stayed within euglycemic range during INI. Peripheral insulin increased after INI & placebo. Epinephrine decreased after INI compared to placebo.	2
Craft et al. (2012) [4]	Randomized, double-blind, placebo-controlled	104 (59 M)	Older adults, 64 aMCI, 40 probable AD	20 IU 40 IU	PET, lumbar puncture, ADAS-Cog, ADAS-ADL scale	INI stabilized/improved cognition, function, & cerebral glucose metabolism for adults with aMCI or AD.	2
Grichisch et al. (2012) [158]	Randomized, open-label, controlled	8 (3 M)	Healthy, 18–34 yo, BMI 20–25	160 IU	Cerebral blood flow, MRI- BOLD response in visual cortex, ASL	No direct INI effects on baseline & stimulus-induced CBF. No change in task-induced BOLD post-INI in visual cortex. No evidence of direct INI effect on CBF.	2
Hallschmid et al. (2012) [159]	Randomized, placebo-controlled, balanced, crossover	13 (0 M)	Female, healthy, 22–24 yo, BMI 21–22, non-smoking	160 IU	Habitual eating behavior, tendency towards disinhibition	INI reduced appetite & snack intake during the postprandial state but not during fasting. Plasma glucose decreased post-INI but remained within euglycemic range. Postprandial INI enhances the satiating effect of meals & reduces palatable snack intake	2
Heni et al. (2012) [160]	Randomized, crossover	103 (35 M)	Healthy, 19–37 yo, BMI 22.6±2.9, no psychiatric, neurologic, metabolic illness	160 IU	Plasma glucose, insulin, C- peptide, fMRI (n12), HOMA-IR	After INI, plasma insulin increased & glucose decreased, increased activity in hypothalamus, putamen, right insula, & OFC. Peripheral insulin sensitivity decreased immediately after INI, & increased 1 hour post-INI.	2
Jauch-Chara et al. (2012) [54]	Randomized, double-blind, placebo-controlled, crossover	15 (M)	Male, healthy, 23–25 yo, BMI 22.2±0.4	40 IU	Brain ATP, PCr, blood glucose & insulin, caloric consumption	Increase in brain ATP 10 minutes post-INI. INI raised PCr content. Plasma glucose was comparable throughout the entire study. C-peptide & insulin were similar at baseline & did not change during the study. INI reduced total caloric consumption by 11.7%.	2
McIntyre et al. (2012) [161]	Randomized, double-blind, placebo-controlled	62 (33 M)	28–51 yo, bipolar disorder 1/2, euthymic	40 IU	Hippocampus-dependent memory recollection tasks, premorbid IQ	INI improved one executive function measure, was well tolerated, no hypoglycemias or safety concerns	2
Brunner et al. (2013) [162]	Double-blind, placebo-controlled, balanced, crossover	17 (10 M)	Healthy, 24.6±0.7 yo, BMI 22±0.4, normosmic	40 IU	Glucose, insulin, cortisol pre & post-INI/placebo. Olfactory threshold testing	Serum insulin & cortisol were not altered after INI. Statistically significant drop in plasma glucose within euglycemic range. Olfactory discrimination skills unaffected in response to INI.	
Claxton et al. (2013) [163]	Randomized, double-blind, placebo-controlled	104 (59 M)	Older adults, 64 aMCI, 40 probable ADC	20 IU 40 IU	PET, lumbar puncture, ADAS-Cog, ADAS-ADL scale	20 IU improved story recall over time compared to placebo. 40 IU improved memory in men, not in women. When comparing INI to placebo, only females benefit from INI. ApoE4 did not predict treatment response for cognitive or functional outcomes. ApoE4- negative males benefited from 40 IU, ApoE4-negative females declined over time on 40 IU.	2
Fan et al. (2013) [164]	Randomized, double-blind, placebo-controlled	45 (36 M)	Adults, 18–65 yo, schizophrenia/schizoaffective disorder, stable antipsychotic dose	20 IU 40 IU 160 IU	PANS & SANS performance	No significant differences in psychopathology, cognitive outcomes, or adverse effects between groups.	2
Li et al. (2013) [165]	Randomized, double-blind, placebo-controlled	39 (32 M)	Adults, 18–65 yo, schizophrenia/schizoaffective disorder, stable antipsychotic dose	160 IU	Body weight, BMI, WC, DXA, fat mass, lean mass, total mass	INI did not affect body weight, BMI, WC, waist-hip ratio, resting energy expenditure, BMC, fat mass, fat %, lean mass, or total mass. No significant differences in fasting glucose, insulin, HOMA-IR, HbA1c, CRP, total cholesterol, LDL, HDL, TG, & LDL. No beneficial effect INI on major metabolic outcomes.	2
Kullmann et al. (2013) [166]	Randomized, placebo-controlled, crossover	17 (0 M)	Female, healthy, 24.4 ± 2.2 yo, BMI 21.1 ± 1.6	160 IU	resting state fMRI, fALFF	INI induced fALFF decrease in hypothalamus & OFC, fasting plasma glucose & insulin did not differ between INI-placebo. Intrinsic brain activity is modulated by INI 30 & 90 minutes after application. BMI-associated activity in response to INI in the PFC & ACC. INI modulated central elements of the reward system in the OFC.	2
Ferreira de Sa et al. (2014) [167]	Randomized, placebo-controlled, balanced	54 (M)	Male, healthy, 19–36 yo	40 IU	Salivary cortisol, EMG blink response, subjective motivation to eat, hunger, stress	Startle responsiveness was not affected by INI.	2
Heni et al. (2014) [168]	Randomized, single-blind, placebo-controlled, crossover	15 (M)	Male, 10: 26 ± 1.3 yo, BMI 21.8 ± 0.7 5: 28 ± 1.7 yo, BMI 33.2 ± 3.7	160 IU	fMRI, hyperinsulinemic-euglycemic insulin clamp, blood glucose, insulin, C- peptide, EKG, HR variability	INI improves insulin sensitivity in lean men. C-peptide & glucose levels did not differ between INI-placebo. Change in high-frequency HR variability after INI. Change in hypothalamic CBF after INI correlated with change in insulin sensitivity. INI does not cause peripheral insulin resistance & enhances whole-body insulin sensitivity.	2
Iwen et al. (2014) [169]	Placebo-controlled, balanced, crossover	14 (M)	Male, healthy, 24.7 ± 1.1 yo; BMI 24.4 ± 0.6	160 IU	FFA, TG, blood glucose, glucoregulatory hormones, BIA, abdominal subcutaneous tissue	INI lowered FFA concentration, TG concentrations unchanged. No treatment effects on blood glucose, insulin, C-peptide, glucagon, ACTH, cortisol, or leptin. Epinephrine, norepinephrine, & TSH unchanged. INI effect was conveyed through CNS pathways.	3
Ketterer et al. (2014) [170]	Randomized, single-blind, crossover	43 (23 M)	40 ± 13 yo, BMI 30.3 ± 9.7	160 IU	MEG, functional connectivity, plasma glucose, insulin, & C-peptide, food-related visual working memory	High brain insulin sensitivity facilitates weight loss during lifestyle interventions. Brain insulin sensitivity determines the effectiveness of lifestyle interventions in terms of weight loss.	2
Novak et al. (2014) [43]	Randomized, double-blind, placebo-controlled, crossover	29 (12 M)	15 healthy, 14 T2DM for >5 years, 50–70 yo	40 IU	Regional perfusion, vasoreactivity, brief visuospatial memory test, verbal fluency	INI does not affect systemic glucose levels, improves visuospatial memory & vasoreactivity in anterior brain regions.	2
Schilling et al. (2014) [56]	Randomized, double-blind, two-by-two, parallel	48 (M)	Male, healthy, 20–27 yo, right-handed	40 IU	Salivary cortisol, mood & hunger ratings, MRI	Increase in regional CBF in insular cortex post-INI.	2
Brunner et al. (2015) [171]	Double-blind, counter-balanced, crossover	18 (M)	Male, healthy, 24.2 ± 0.8 yo BMI: 22.6 ± 0.4, normosmic	40 IU	Odor-place memory, odor-recognition, pleasantness ratings, blood glucose, insulin, epinephrine, cortisol, leptin, & acetylcholine levels	INI eases odor-cued delayed recall of spatial memories. INI did not exert relevant systemic effects on glucose & insulin levels. INI may have a global impact on CNS networks relevant for odor-cued recall of spatial memory.	3
Dash et al. (2015) [59]	Randomized, single blind, crossover	8 (M)	Male, healthy, 49.1 ± 2 yo, BMI 23.9 ± 0.8	40 IU	Plasma glucose, insulin, FFA, TG	Transient decrease in glucose & transient increase in insulin post-INI. INI suppresses endogenous glucose production compared to placebo.	2
Gancheva et al. (2015) [172]	Randomized. placebo-controlled, single-blind, crossover	20 (16 M)	10:healthy, 24–27 yo, BMI 22–24; 10: insulin-naïve T2DM on oral glucose lowering agents, 58–62 yo, BMI 28–30	160 IU	MRS, plasma TG, FFA, cholesterol, glucose, insulin, C-peptide, HbA1c, AST, ALT, HOMA-IR, QUICKI	INI did not affect glucose production; increased hepatic ATP, decreased hepatic TG in healthy group, not in T2DM. Transient insulin increase after INI, transient glucose decline in glucose & FFA	2
Kullmann et al. (2015) [83]	Randomized, placebo-controlled, crossover	48 (27 M)	Adults, 25 lean,10 overweight, 13 obesity, BMI 19–46, no psychiatric, neurologic or metabolic diseases	160 IU	MRI, CBF	After INI, hypothalamic CBF decreased in lean, overweight, & obese participants. INI reduced CBF in prefrontal cortex of lean participants only, which correlated with peripheral insulin sensitivity, disinhibition, & food craving. Magnitude of response correlated with visceral adipose tissue. INI reduced sweet food craving in lean men only.	2
Schopf et al. (2015) [173]	Open label, crossover	10 (7 M)	22–56 yo, BMI 21.5–36.3, post-infectious olfactory loss	40 IU	Olfactory detection test	Improved olfactory sensitivity after INI; improved odor identification in subjects with higher BMI.	3
Zhang et al. (2015) [41]	Randomized, double-blind, placebo-controlled	28 (11 M)	14 T2DM, 14 Control, 50–70 yo	40 IU	Resting state fMRI, neuropsychological assessment	Single INI dose increases resting-state functional connectivity between hippocampal regions & default mode network in older adults with T2DM. Increased resting-state connectivity between hippocampal regions & medial frontal cortex after INI compared to placebo.	2
Brunner et al. (2016) [57]	Double-blind, placebo-controlled, counter-balanced	11 (M)	Male, healthy, 24.9 ± 1.3 yo, BMI 23.7 ± 0.2, right- handed, non-smoking	40 IU	fMRI, memory performance (encoding maze with visual & olfactory clues)	INI has no effect on declarative memory; INI application is sensitive to methodological variations.	3
Feld et al. (2016) [174]	Double-blind, placebo-controlled, balanced, crossover	32 (16 M)	Healthy, 18–30 yo, BMI ≥ 26, non-smoking	160 IU	Declarative memory, blood glucose, GH, insulin, EEG, vigilance, sleepiness, mood, hunger, thirst	INI increased GH concentrations in the first night.	3
Zwanenburg et al. (2016) [175]	Randomized, double-blind, placebo-controlled, stepped- wedge	25 (6 M)	Children, 1–16 yo, starting weight 25.3 ± 13.8 kg, confirmed 22.q13.3 deletion including SHANK3 gene	20–40 IU/day (age-dependent)	Cognitive, language, motor development, adaptive, social, & emotional behavior	INI did not cause serious AE, increased developmental functioning level by 0.4–1.4 months per 6-month period, & had a significant effect for cognitive & social skills for children > 3 years.	2
Akintola et al. (2017) [42]	Randomized, double-blind, placebo-controlled, crossover	19 (M)	Male, 11: 60–69 yo, 8: 20–26 yo	40 IU	CBF & perfusion, venous glucose & insulin	INI improved tissue perfusion of occipital cortex & thalamus in older adults only. INI did not change mean blood flow through cerebropetal arteries.	2
Cha et al. (2017) [176]	Randomized, double-blind, placebo-controlled, crossover	35 (13 M)	18–65 yo, major depressive disorder (DSM-IV)	160 IU	MADRS Positive & Negative Affect Schedule	INI did not improve overall mood, emotional processing, neurocognitive function, or self-reported quality of life.	2
Craft et al. (2017) [44]	Randomized, double-blind, placebo-controlled	36 (17 M)	22 aMCI, 14 probable AD, 60–80 yo, (MMSE<15)	40 IU	Delayed list & story recall composite score, global cognition, daily functioning, MRI volume changes, CSF AD markers	Group on regular INI had better memory after 2 & 4 months compared to placebo. No effects observed in INI-Detemir group.	2
Heni et al. (2017) [177]	Randomized, placebo-controlled	21 (M)	Male, healthy, 23–29 yo, 10 lean, BMI 23.3 ± 1.8; 10 overweight, BMI 28.3 ± 4.6	160 IU	Endogenous blood glucose production rate, CBF, fMRI	Brain insulin may improve peripheral insulin sensitivity. INI administration to the brain did not alter peripheral metabolism in overweight participants.	2
Kullmann et al. (2017) [178]	Randomized, single-blind, placebo-controlled, crossover	47 (26 M)	Adults, 25 lean, 10 overweight, 12 obesity, 22–29 yo, BMI 19–40	160 IU	fMRI, functional connectivity, total body adipose tissue, visceral adipose tissue, peripheral insulin sensitivity index	INI increases functional connectivity between prefrontal regions of default mode network, hippocampus, & hypothalamus. Change in hippocampal functional connectivity significantly correlated with visceral adipose tissue & change in subjective hunger feelings after INI.	2
Kullmann et al. (2017) [179]	Open label, crossover	48 (27 M)	Healthy, 21–40 yo, BMI 19.2–46.5	160 IU	MRI CBF, 75 gr OGTT, plasma glucose, insulin, & C- peptide, insulin sensitivity indexes	Hypothalamic insulin resistance might contribute to pancreatic insulin hypersecretion.	3
Rodriguez-Raecke et al. (2017) [180]	Pseudo-randomized, placebo-controlled	24 (M)	Male, healthy, 25 ± 4.7 yo, BMI range 19.6–26.8	40 IU	Insulin, glucose, leptin, HOMA-IR, Beck depression inventory, MOCA, Brief Symptom Inventory	INI improved taste sensitivity for sweet & salty tastes.	3
Santiago & Hallschmid (2017) [181]	Double-blind, placebo-controlled, balanced, crossover	51 (26 M)	32 healthy, 23.7 ± 0.4 yo, 19 70.8 ± 0.8 yo, BMI 22.8 ± 0.3	160 IU	Memory test battery, blood glucose, EEG, HR	INI before sleep reduced carbohydrate intake by 9%, did not alter hunger, thirst, or fatigue before breakfast. INI did not alter sleep latency or whole-night sleep architecture.	3
Scherer et al. (2017) [55]	Randomized, double-blind, placebo-controlled	20 (M)	Male, healthy, 26–40 yo, BMI 23.9–25.9	160 IU	Hepatic TG content & circulating BCAA	INI did not alter body weight, BMI, or hepatic lipid contents; but reduced circulating BCAA levels.	2
Xiao et al. (2017) [58]	Randomized, single-blind, placebo-controlled, crossover	9 (M)	Male, healthy, 45–51 yo, BMI 25.4–26.6, normolipidemic, normoglycemic	40 IU	Plasma TG, TG rich lipoprotein, plasma FFA	INI does not modulate hepatic & intestinal lipoprotein particle production.	2
Thienel et al. (2017) [182]	Double-blind, placebo-controlled, crossover	44 (24 M)	14 healthy 67–74 yo, BMI 24.8 ± 0.6; 30 healthy 19–30 yo, BMI 22.9 ± 0.3	160 IU	Serum cortisol, C-peptide, insulin, glucose, plasma ACTH, appetite, thirst, sleepiness, well-being, subjective sleep quality	Compared to placebo, INI decreased cortisol in elderly subjects during the first half of the night. Insulin was not affected by INI in the elderly. Insulin rose shortly after INI in young subjects. C-peptide decreased after INI in both groups. INI did not alter sleep latency, whole night sleep architecture or total sleep time.	3
van Opstal et al. (2017) [183]	Randomized, double-blind, placebo-controlled, crossover	8 (M)	Male, healthy, 22.3 ± 1.8 yo, BMI 23.6 ± 2.2	40 IU	Hypothalamic activation, BOLD	INI did not change circulating glucose or insulin, it further decreased post-glucose hypothalamic BOLD response. In healthy volunteers, higher plasma glucose lead to reduced hypothalamic BOLD responses. In patients with T2DM, there was no post-glucose decrease in BOLD response.	2
Hamidovic et al. (2018) [184]	Randomized, placebo-controlled, parallel	50 (37 M)	18–65 yo, BMI 18.5–30, smoking, normal vital signs, blood glucose	60 IU	Learning, episodic memory, immediate & delayed recall	INI did not improve learning, short or long-term recall.	2
Kullmann et al. (2018) [94]	Randomized, placebo-controlled, crossover	9 (M)	Male, healthy, 23–30 yo, BMI 20–26	40 IU 80 IU 160 IU	fMRI, HR variability, plasma insulin, C-peptide, glucose, subjective hunger	INI dose-dependently modulates regional brain activity, strongest effects after 160 IU. 160 IU transiently increases circulating insulin concentrations.	2
Ritze et al. (2018) [185]	Randomized, placebo-controlled, crossover	36 (M)	Male, healthy, 18–40 yo, BMI 23.5 ± 0.3	160 IU	Body weight, body composition, glucose, insulin, ACTH, cortisol, GH, IGF-1, adiponectin, leptin, declarative & procedural memory	INI did not induce changes in body weight or body composition, delayed word recall improved after INI evening administration, serum cortisol concentrations reduced after 2 weeks INI.	2
Rodriguez-Raecke et al. (2018) [142]	Double-blind, placebo-controlled, crossover	30 (16 M)	Healthy, 22–26 yo, BMI<25, non-smoking, normosmic	40 IU	Olfactory sensitivities for n- butanol & peanut, blood glucose, insulin, leptin, cortisol	After INI, female olfactory sensitivity for n-butanol was lower. Effects of cortical insulin levels are likely gender-modulated.	3
Wingrove et al. (2019) [61]	Double-blind, placebo-controlled, crossover	16 (M)	Males, healthy, 20–28 yo, BMI 25–31	160 IU	CBF, ASL, blood glucose, insulin, C-peptide	Significant decrease in regional CBF in areas with high insulin-receptor density after INI compared to placebo. No changes in blood glucose, insulin, or C-peptide.	2
Craft et al. (2020) [46]	Randomized, double-blind, placebo-controlled	289 (155 M)	289 (155 M)55–85 yo, mild cognitive impairment/AD, (MMSE>20)	40 IU	Mean score change on ADAS-COG	No cognitive or functional benefits were observed with INI over a 12-month period.	2

* Evidence level determined in accordance with CEBM levels of evidence. Studies were classified as Level 2 evidence if they mentioned random treatment allocation in their study design or if observational with a dramatic effect; Level 3 if they were non-randomized controlled studies & Level 4 if they were presented as case reports or case-series. Table 2 summarizes clinical trials using INI & the available evidences for nose-to-brain delivery.

ACC: Anterior Cingulate Cortex; ACTH: Adrenocorticotropic Hormone; AD: Alzheimer Disease; ADAS-ADL: Alzheimer’s Disease Assessment Scale-Activities of Daily Living; ADAS-Cog: Alzheimer’s Disease Assessment Scale-Cognitive; AE: Adverse Effects; AERP: Auditory Evoked Related Potentials; ALT: Alanine Aminotransferase; aMCI: Amnestic Mild Cognitive Impairment; ApoE: Apolipoprotein E; ASL: Arterial Spin Labeling; AST: Aspartate Aminotransferase; ATP: Adenosine Triphosphate; BCAA: Branched-Chain Amino Acid, BIA: Body Impedance Analysis; BMC: Bone Mineral Content; BMI: Body Mass Index; BOLD: Blood Oxygenation Level Dependent; BSI: Body Sensitivity Index; BP: Blood Pressure; CBF: Cerebral Blood Flow; CRP: C-Reactive Protein; CSF: Cerebrospinal Fluid; DAD: Disability Assessment in Dementia Questionnaire; DBP: Diastolic blood pressure; DM: Diabetes Mellitus; DSM-IV: Diagnostic & Statistical Manual of Mental Disorders 4th edition; DXA: Dual- energy X-Ray Absorptiometry; EEG: Electroencephalogram; EMG: Electromyography; FALFF: Fractional Amplitude of Low Frequency Fluctuations; FFA: Free Fatty Acids; fMRI: functional Magnetic Resonance Imaging; GH: Growth Hormone; HbA1c: Hemoglobin A1c; HOMA-IR: Homeostatic Model Assessment Insulin Resistance; HPA: Hypothalamic-Pituitary-Adrenal; IGF-1: Insulin-like Growth Factor 1; INI: Intranasal Insulin; IU: International Units; GDS: Geriatric Depression Scale; HDL: High Density Lipoprotein; HR: Heart Rate; LDL: Low Density Lipoprotein; MADRS: Montgomery Asberg Depression Rating Scale; MEG: Magnetoencephalography; MMSE: Mini Mental Status Examination; MRS: Magnetic Resonance Spectroscopy; MOCA: Montreal Cognitive Assessment; PANS: Positive & Negative Syndrome Scale; OGTT: Oral Glucose Tolerance Test; OFC: Orbitofrontal Cortex; PCr: Phosphocreatine; PET: Positron Emission Tomography; PFC: Prefrontal Cortex; SANS: Scale for Assessment of Negative Symptoms; SBP: Systolic blood pressure; QUICKI: Quantitative Insulin Sensitivity Check Index; T2DM: Type 2 Diabetes Mellitus; TG: triglycerides; WC: Waist Circumference; WMS-RLM: Wechsler Memory Scale Revised Logical Memory; Yo: Years old

**Table 3:** Clinical trials evaluating nose-to-brain delivery of substances other than insulin.

Substance	Author (Year)	Design	N (Male)	Characteristics	Dose	Outcome measures	Conclusion	Evidence level*
CCK	Pietrowsky et al. (1996) [102]	Double-blind, placebo-controlled, crossover	20 (10 M)	Healthy, 21–27 yo, mean weight 69.5 kg, non- smoking, no hearing deficiency	10 μg	AERP while performing an attention task; plasma ACTH & cortisol	P3 complex significantly increased only after IN-CCK. IN-CCK increased ACTH when compared to placebo, cortisol did not differ. Plasma CCK was comparable after IN & IV administration.	3
CCK	Pietrowsky et al. (2001) [103]	Double-blind, placebo-controlled, crossover	32 (16 M)	Healthy, 22–38 yo, mean BMI 21.9	10 μg	AERP while performing an attention task; plasma ACTH & cortisol AERP, plasma CCK	After IN-CCK, post-stimulus AERP latency interval increased in women compared to placebo.	2
CCK	Denecke et al. (2002) [101]	Double-blind, crossover	20 (10 M)	Healthy, 21–38 yo, non- smoking	10 μg 20 μg	AERP, BP, HR, salivary cortisol	Both doses of IN-CCK increased LPC magnitude compared to placebo. No change in BP, HR, or salivary cortisol.	3
CCK	Smolnik et al. (2002) [100]	Double-blind, placebo-controlled	26 (14 M)	13 PD, 63–71 yo, without dementia, continuous L-dopa therapy for 6 months; 13 age-matched controls	25 μg	AERP, UPDRS-III, fine motor skills	IN-CCK delayed peak latency of N2 & P3 AERP components in PD & reduced them in controls. IN-CCK reduced peak latency. No difference in fine motor skills after IN-CCK.	3
CCK	Denecke et al. (2004) [104]	Double-blind, placebo-controlled, crossover	16 (0 M)	Females, healthy, 20–28 yo, non-smoking, non-pregnant	10 μg	AERP, BP, HR, salivary cortisol, plasma CCK, alertness task	P3 amplitude largest following IN-CCK. BP, HR, salivary cortisol, & plasma CCK did not differ. Alertness not affected by IN-CCK.	3
CCK	Schneider et al. (2005) [98]	Randomized, double-blind, placebo-controlled, between-subject	64 (32 M)	Healthy, 18–39 yo, non- smoking	40 μg	Conscious & unconscious memory performance	IN-CCK decreases controlled memory recollection component	2
CCK	Schneider et al. (2009) [99]	Randomized, double-blind, factorial	64 (32 M)	Healthy, 20–39 yo	40 μg	Conscious & unconscious memory performance, self-perceived activation levels	CCK increased familiarity-based recognition memory.	2
EPO	Santos-Morales et al. (2017) [110]	Randomized, open-label, parallel	25 (11 M)	Healthy, 18–40 yo	1 mg 0.5 mg	Baseline health change, CBC, coagulation parameters, glycemia, creatinine, urea, liver enzymes	IN-EPO is safe, well tolerated, did not stimulate erythropoiesis.	2
EPO	Pedroso et al. (2018) [186]	Randomized, placebo-controlled	26 (15 M)	45–67 yo, PD, Hoehn & Yahr stage 1–2	1 mg	Global cognitive & executive function, visual memory	IN-EPO improves cognitive function in PD.	2
Melanocortin	Fehm et al. (2001) [111]	Randomized, placebo-controlled, crossover	36 (18 M)	Healthy,19–35 yo, BMI 21.9 ± 0.3, non-smoking	0.5 mg MSH/ACTH4-10 & 0.84 mg deacetyl-α-MSH	BIA, plasma leptin, insulin, ACTH, cortisol, TSH, T3, T4, BP, serum electrolytes, creatinine, CRP, liver enzymes	6-week treatment decreased body fat & weight. Body fat reduction associated with a decrease in plasma leptin & insulin. Cardiovascular parameters, cortisol, thyroid & laboratory measures unchanged.	2
Melanocortin	Born et al. (2002) [88]	Open label	36 (27 M)	Healthy, 25–41 yo	10 mg	CSF	Increased CSF melanocortin within 10 minutes of IN administration, peaked at 30 minutes, & remained elevated at 80 minutes. No change in plasma MSH/ACTH concentration.	2
Melanocortin	Wellhöner et al. (2012) [114]	Randomized, double-blind, crossover	10 (M)	Male, healthy, 25–30 yo, BMI 20–25, stable weight for 3 months	10 mg	Interstitial glycerol, local blood flow, BP, HR, FFA, superficial peroneal nerve activity	IN-MSH-ACTH increases white adipose tissue lipolysis & muscle sympathetic nerve activity.	2
Glutathione	Mischley et al (2013) [118]	Survey	70 (19 M)	20–78 yo, on IN glutathione	NA	Individual tolerability perception, adverse events, health benefits	Well-tolerated, 78% reported positive overall experience; 45% symptom improvement, 28% improved sense of well-being, 27% decreased sinus infection incidence.	N A
Glutathione	Mischley et al. (2015) [117]	Randomized, double-blind, placebo-controlled	30 (15 M)	>21 yo, PD diagnosed in last decade	600 mg	UPDRS, CBC, liver enzymes, BUN, creatinine	IN glutathione is safe & well tolerated. Mild improvement in UPDRS.	2
Glutathione	Mischley et al. (2016) [116]	Open label	15 (11 M)	Adults, 54–76 yo, PD, Hoehn & Yahr stage 2–3	200 mg	MRS	IN-GSH raises brain GSH levels.	4
Glutathione	Mischley et al. (2017) [119]	Randomized, double-blind, placebo-controlled	45 (23 M)	Adults, 49–71 yo, PD diagnosed in last decade	100 mg 200 mg	UPDRS, GSH tolerability, MR spectroscopy	200 mg group improved UPDRS. IN-GSH was not superior to placebo.	2
Perillyl alcohol	DaFonseca et al. (2006) [123]	Case report	1 (0 M)	Female, 62 yo, anaplastic oligodendroglioma, Karnofsky index ≥70%	220 mg	Tolerability, tumor size	No toxicity evidence after 5 months therapy, decreased tumor size.	4
Perillyl alcohol	DaFonseca et al. (2008) [126]	Open label	37 (NA)	35–69 yo, relapsing malignant glioma, Karnofsky index ≥70%, measurable contrast enhancing tumor on MRI	220 mg	Disease progression, progression-free survival, tolerability	Improved tumor response rates after treatment; overall treatment was well tolerated.	3
Perillyl alcohol	DaFonseca et al. (2011) [125]	Open label, controlled	89 (50 M)	>18 yo, recurrent GBM, measurable contrast- enhancing tumor on MRI, Karnofsky index ≥70%, no laboratory abnormalities, CHF evidence or unstable angina.	440 mg	Overall survival, tumor recurrence, clinical progression	IN-POH increased survival in patients with primary & secondary GBM localized to deep brain regions.	3
Perillyl alcohol	DaFonseca et al. (2013) [124]	Retrospective cohort	185 (NA)	154 GBM, 26 grade 3 astrocytoma, 5 anaplastic oligodendroglioma	266.8 mg 533.6 mg	Long-term response, toxicity	19% remain in remission after 4 years, IN-POH is safe & efficient for recurrent malignant glioma	2
Perillyl alcohol	Faria (2020) [187]	Retrospective cohort	100 (62 M)	18–78 yo; recurrent GBM, measurable contrast enhancing tumor on MRI, failed conventional therapy	NA	Presence of MTHFR rs1801133 variant	IN-POH prolonged survival of patients with recurrent GBM. IN-POH decreases deleterious effects of global DNA hypomethylation due to mutated MTHFR variant.	3
Angiotensin II	Derad et al. (1998) [127]	Blinded, balanced, placebo-controlled, crossover	10 (M)	Male, healthy, 22.8–28.8 yo, 68.6–84.6 kg, non- smoking	100 μg 400 μg	Plasma AGII, vasopressin, catecholamines, BP, activation feelings, mood	Low-dose IV & IN-ANGII did not affect plasma AGII, vasopressin, norepinephrine, BP, mood, or activation feelings. Plasma vasopressin & norepinephrine increased significantly after high dose IN-ANGII, mirroring intraventricular ANGII administration.	2
Angiotensin II	Derad et al. (2014) [130]	Double-blind, placebo-controlled, balanced, crossover	16 (8 M)	Healthy, 21–27 yo, normotensive, non-smoking	400 μg	Plasma ANGII, aldosterone, renin, vasopressin, norepinephrine, continuous BP & HR recordings	Plasma ANGII increased after administration & remained elevated for 95 minutes. Systolic BP significantly decreased after IN-AGII compared to placebo. Other measured hormones did not change significantly.	2
Neurotrophic factors	De Bellis et al. (2018) [139]	Case series	4 (0 M)	Female, 58–64 yo, with FTD & CBS	10 μl	Cognition, rigidity, speech, PET	Long-term IN-NGF improved motor & cognitive abilities. Significant increase in FDG uptake after 3 months of IN-NGF.	4
Neurotrophic factors	Chiaretti et al. (2017) [140]	Case report	1 (M)	Male, 4 yo, post-TBI, unresponsive wakefulness syndrome	0.1 mg/kg	Sensorimotor score, SPECT/CT, EEG, VEP, CSF	Improved communication strategy, attention, verbal comprehension, facial mimicry, head rotation, oral motility, bowel function & cough reflex after IN-NGF. Increased FDG uptake in cortical, subcortical regions, & CSF after treatment.	4
Sumatriptan	Luthringer et al. (2009) [144]	Randomized, open-label	12 (1 M)	Healthy, 21–43 yo, BMI 18– 24, migraine without aura	10 mg 20 mg	EEG, sumatriptan plasma concentration, subjective migraine assessment	IN sumatriptan induced a similar EEG profile than SC sumatriptan.	2
Sumatriptan	Djupesland et al. (2010) [65]	Randomized, double-blind, placebo-controlled, parallel	117 (17 M)	18–65 yo, moderate-severe migraine, within 4 hours of onset	10 mg 20 mg	Headache severity score, functional disability, migraine-associated symptoms, EKG, CBC	More subjects on sumatriptan had symptom resolution at 60 & 120 minutes compared to placebo.	2

* Evidence level determined in accordance with CEBM levels of evidence. Studies were classified as Level 2 evidence if they mentioned random treatment allocation in their study design or if observational with a dramatic effect; Level 3 if they were non-randomized controlled studies & Level 4 if they were presented as case reports or case-series.

ACTH: Adrenocorticotropic Hormone; AERP: Auditory Event Related Potential; AGII: Angiotensin II; BIA: Body Impedance Analysis; BMI: Body Mass Index; BP: Blood pressure; BUN: Blood Urea Nitrogen; CBC: Complete Blood Count; CBS: Cortico-Basal Syndrome; CCK: Cholecystokinin; CHF: Congestive Heart Failure; CRP: C- Reactive Protein; CSF: Cerebrospinal Fluid; DHE: Dihydroergotamine Mesylate; EEG: Electroencephalography; ERP: Event Related Potential; EPO: Erythropoietin; FDG: Fluorodeoxyglucose; FTD: Fronto-Temporal Dementia; GBM: Glioblastoma Multiforme; HR: Heart Rate; IN: Intranasal; IV: Intravenous; LPC: Late Positive Complex; mg: milligram; MRS: Magnetic Resonance Spectroscopy; MSH: Melanocyte Stimulating Hormone; NGF: Nerve Growth Factor; PD: Parkinson Disease; PDP: Process Dissociation Procedure; TBI: Traumatic Brain Injury; TSH: Thyroid Stimulating Hormone; SC: Subcutaneous; SPECT-CT: Single Photon Emission Computed Tomography; UPDRS-III: Unified Parkinson Disease Rating Scale; VEP: Visual Evoked Potentials; Yo: Years old; μg: micrograms

## ABBREVIATIONS

AUC: Area Under the Curve
BBB: Blood Brain Barrier
CCK: Cholecystokinin
CNS: Central Nervous System
CSF: Cerebrospinal Fluid
CEBM: Centre for Evidence-Based Medicine
EPO: Erythropoietin
INI: Intranasal Insulin
IU: International Units
MemAID: Memory Advancement by Intranasal Insulin in Type 2 Diabetes
MRI: Magnetic Resonance Imaging
MRS: Magnetic Resonance Spectroscopy
MSH/ACTH4–10: Melanocyte-Stimulating Hormone/Adrenocorticotropin 4–10
PD: Parkinson Disease
PET: Positron Emission Tomography
RCT: Randomized Controlled Trial
SNIFF: Study of Nasal Insulin to Fight Forgetfulness
SPECT: Single-Photon Emission Computed Tomography
